# An Atomistic-Scale Study for Thermal Conductivity and Thermochemical Compatibility in (DyY)Zr_2_O_7_ Combining an Experimental Approach with Theoretical Calculation

**DOI:** 10.1038/srep21232

**Published:** 2016-02-18

**Authors:** Liu Qu, Kwang-Leong Choy, Richard Wheatley

**Affiliations:** 1 UCL Institute for Materials Discovery, University College London, London, WC1E 6BT, UK; 2 Department of Chemistry, University of Nottingham, Nottingham, NG7 2RD, UK

## Abstract

Ceramic oxides that have high-temperature capabilities can be deposited on the superalloy components in aero engines and diesel engines to advance engine efficiency and reduce fuel consumption. This paper aims to study doping effects of Dy^3+^ and Y^3+^on the thermodynamic properties of ZrO_2_ synthesized via a sol-gel route for a better control of the stoichiometry, combined with molecular dynamics (MD) simulation for the calculation of theoretical properties. The thermal conductivity is investigated by the MD simulation and Clarke’s model. This can improve the understanding of the microstructure and thermodynamic properties of (DyY)Zr_2_O_7_ (DYZ) at the atomistic level. The phonon-defect scattering and phonon-phonon scattering processes are investigated via the theoretical calculation, which provides an effective way to study thermal transport properties of ionic oxides. The measured and predicted thermal conductivity of DYZ is lower than that of 4 mol % Y_2_O_3_ stabilized ZrO_2_ (4YSZ). It is discovered that DYZ is thermochemically compatible with Al_2_O_3_ at 1300 °C, whereas at 1350 °C DYZ reacts with Al_2_O_3_ forming a small amount of new phases.

High temperature ceramic materials such as 6–8 wt. % Y_2_O_3_ stabilized ZrO_2_ (6–8 wt. % YSZ, i.e. 3–4.5 mol % YSZ) have been investigated and used in extremely severe environments such as the thermal barrier coatings (TBCs) on turbine blades^1^. 6–8 wt. % YSZ coatings deposited on turbine blades, can improve the application temperature and the performance of engines for propulsion in aviation, automotive and marine industries and power generative system^2^,^3^. The deposited ceramic TBCs on superalloy components can lower the surface temperature and increase the durability of the components, which advances the engine efficiency and brings about environmentally friendly effects such as low emission and fuel consumption. However, the phase instability and sintering effects of conventional 6–8 wt. % YSZ coatings above 1200 °C, which result in reduced durability and reliability, have hindered further application in TBCs^4^.

Various ceramic materials are developed to satisfy the requirements of new TBCs including low thermal conductivity, high coefficient of thermal expansion (CTE), thermochemical stability, and sintering resistance^5^,^6^. Promising compositions such as Re_2_Zr_2_O_7_, La_2_Ce_2_O_7_, LaMgAl_11_O_19_, quasi-eutectoid (La_1-x_Yb_x_)_2_Zr_2_O_7_, and Y_4_Al_2_O_9_ which are reported to exhibit improved thermomechanical properties, are considered as potential candidates for new TBCs^7^,^8^,^9^,^10^,^11^. The nonstoichiometry pyrochlore Nd_2-x_Zr_2+x_O_7+x/2_ shows better thermomechanical property than 4YSZ^12^. 5 to 20 mol % Dy_2_O_3_ doped 5YSZ has been investigated by Wang^13^, who summarized that air-plasma-sprayed coatings exhibited the monoclinic and cubic structures and 10 mol % (Dy, Y)-ZrO_2_ coatings had the highest resistance to heat transfer. It is reported that Dy-YSZ is composed of the cubic/tetragonal structure at lower doping level, and the thermal conductivity decreases with the addition of Dy_2_O_3_ and Y_2_O_3_ to ZrO_2_^14^. However, there is a paucity of studies on the thermodynamic properties of the DYZ system at the atomistic level. Furthermore, the sol-gel approach is effective in controlling the chemical composition and particle morphology, in which case intrinsic material properties can be determined.

MD simulation can be performed to systematically predict the theoretical material properties of various chemical compositions^15^. It involves the calculation of the position and momentum of trajectories of particles based on Newton’s second law of motion^16^. MD simulation enables nano-size models consisting of several million particles and microseconds of real-time to be studied, in contrast to various other modelling approaches^17^. Thermodynamic properties, such as transport properties and vibrational frequencies can be predicted from the time-dependent trajectories of atoms in an MD simulation system^18^. In the direct non-equilibrium MD simulation method, a perturbation is applied to the simulation cell, and the response of the system, such as the heat flux, is measured, from which basis thermal conductivity can be determined^19^.

There is limited information on the thermodynamic properties of DYZ, while no reports exist on the MD simulation of DYZ. This paper emphasizes the development of novel ionic oxide materials via experiment and theoretical calculation. Doping ZrO_2_ with Dy^3+^ which has a higher atomic weight and larger ionic radius than Zr^4+^ can potentially modify the thermal conductivity. The relationship between chemical composition, microstructure and thermal conductivity is studied. The phonon scattering processes which significantly influence the thermal conductivity are highlighted by the theoretical calculation based on the MD model and Clarke’s model. We have investigated the thermochemical compatibility of DYZ with Al_2_O_3_ by the structural characterization and the local chemical environment study of powder mixtures. This present work improves the understanding of the microstructure and thermodynamic properties DYZ on the atomistic level.

## Experimental

### Processing Routes

The starting materials consist of metal salts of Y^3+^ and Dy^3+^, 1-propanol, zirconium propoxide in 1-propanol and acetylacetone. These precursors were mixed to form a solution with the desired molar ratio under argon atmosphere to prevent the fast hydrolysis of Zr(OPr)_4_ with H_2_O. The molar ratio: [AcAc]/[Zr(OPr)_4_] = 0.7. The concentration of Zr(OPr)_4_ was 0.5 mol/L. Deionized water was added to the 1-propanol to achieve the concentration of 10 mol/L. The water in 1-propanol was mixed with the Zr(OPr)_4_ solution with the molar ratio [H_2_O]/[Zr(OPr)_4_] = 8.5. The mixed solution was stirred mechanically for 30 min. The homogeneous sol was dried at 50 °C for several hours to obtain the gel, followed by calcination at 950 °C for 3 hours to obtain ceramic powders. The as-sintered powders were treated at 1200 °C for 2 hours, followed by uniaxial compaction of the powders into pellets. The pellets were sintered at 1200 °C for 6 hours.

### Thermochemical Compatibility

The thermodynamic stability between the as-synthesized DYZ powders and Al_2_O_3_ was studied using a solid-state reaction method. The as-synthesized powders were mixed with Al_2_O_3_ in an equal mass ratio, followed by ball milling for 8 hours. The ball milled powders were sintered at 1300 °C and 1350 °C under air atmosphere to study the thermochemical compatibility.

### Characterization

The crystal structure was characterized via X-ray diffraction (XRD) using a Siemens D500 X-ray diffractometer (Cu-Kα radiation, *λ* = 1.54 Å). Raman analysis was conducted via a Horiba Jobin-Yvon LabRAM spectrometer equipped with an Olympus BX41 microscope (He-Ne laser source, 632.8 nm). The surface morphology and microstructure of the particle and pellets were examined by a Phillips XL30 scanning electron microscope (SEM). The surface chemical analysis was carried out via a Thermo Scientific K-Alpha X-ray photoelectron spectrometer (XPS) using a monochromated Al-Kα X-ray source (*hν* = 1486.6 eV). The thermal conductivity of the pellets was measured using a thermal conductivity analyser (C-Therm TCi) at room temperature. The coefficient of thermal expansion is measured using a TA Q400 Thermomechanical Analyser (TMA) from room temperature to 900 °C under a N_2_ atmosphere with a heating rate of 5 °C/min. The bulk density (*ρ*) was determined by measuring the weight (*w*) and the volume (*v*) of the pellet directly, where *ρ* = *w*/*v*. The theoretical density was estimated via the lattice parameters from XRD, and the molecular weight was calculated based on each molecular formula. The bulk thermal conductivity was calculated based on the porosity and the measured thermal conductivity using,


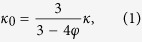


where *φ* is the porosity, *κ*_0_ is the corrected thermal conductivity for fully dense materials, and *κ* is the measured thermal conductivity^20^. The correction is based on the assumption that spherical pores are homogeneously distributed in the medium and the thermal conductivity for pores is infinite.

### Simulation Method

The simulation is conducted based on the rigid-ion model. The simulation cell contains 6 × 6 × 32 crystallographic unit cells, and thermal conductivity is calculated via the non-equilibrium MD simulation method^21^. In a simulation cell, particles such as Y^3+^ ions, Zr^4+^ ions and Dy^3+^ ions are randomly located in cation sites and O^2−^ ions are randomly located in anion sites. The starting positions of 4YSZ are initialized in a tetragonal lattice with an initial displacement, whereas DYZ has zero displacement in the initialized tetragonal lattice. The crystal structure information used for the starting positions is determined via the XRD method. The simulation process consists of an (N, P, T)-ensemble for the system to reach equilibrium and an (N, V, E)-ensemble for the calculation of thermodynamic properties. In the initial (N, P, T)-ensemble, in which the temperature, pressure and number of particles are constant, the atoms reach equilibrium during a period of 5 × 10^4^−10 × 10^4^ time steps (25–50 ps). In the (N, V, E)-ensemble, which is referred to as the system with a constant volume, constant energy and constant number of particles, a temperature perturbation is generated within the simulation cell along the long dimension of the simulation cell during a period of 2.5 × 10^5^ time steps (125 ps). The potential is determined via Buckingham potential for the short-range interaction between ions and the long-range Coulomb interaction.


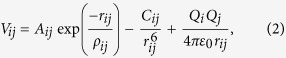


where *A*_*ij*_*, ρ*_*ij*_ and *C*_*ij*_ are Buckingham potential parameters which consist of the Born-Mayer potential, 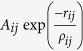 and a van der Waals potential, 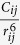. *A*_*ij*_ indicates the repulsion between two ions when their electronic clouds overlap. *ρ*_*ij*_ is the ionic radii of two ions, and *C*_*ij*_ represents the strength of the van der Waals interaction of two ions. *Q*_*i*_*, Q*_*j*_ and *r*_*ij*_ describe the Coulomb interaction. *Q*_*i*_ and *Q*_*j*_ are the charges of two ions, *r*_*ij*_ is the separation of two ions and *ε*_0_ is the vacuum permittivity^22^,^23^. Short-range forces are calculated via the direct summation in which a cut-off radius is applied, and a long-range correction is added. The long-range forces are determined via the Wolf method^24^,^25^. The temperature and the pressure in the (N, P, T)-ensemble are controlled by the Berendsen barostat method and the Anderson thermostat method^26^,^27^. The potential parameters that have been used in the simulation are presented in Table 1.

**Figure 1:**
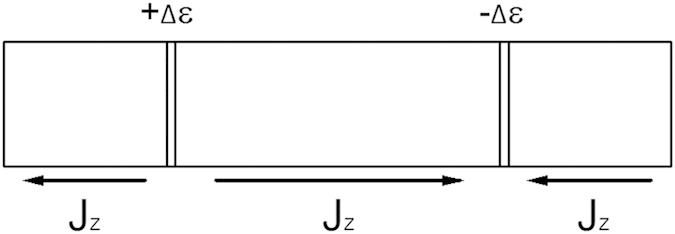
Diagram of the heat conduction in a periodic simulation box (∆ε is the heat incorporated and removed from two slabs).

In the non-equilibrium MD model, the heat is incorporated into a slab of materials and removed from another slab of materials, as shown in Fig. 1. The velocity-rescaling method is applied to the particles within heated and cooled slabs in the (N, V, E)-ensemble, resulting in a net kinetic energy difference of Δ*ε*^22^. The heat current of the periodic simulation cell can be determined by,


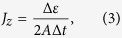


where Δ*ε* is the kinetic energy, Δ*t* is the MD time step (0.5 fs), and *A* is the cross-sectional area^22^. In the MD simulation, the thermal plate power 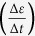 is 320 nW. The simulation cell is divided into 20 slices from which the temperature gradient of the system can be determined, and the thermal conductivity is computed via Fourier’s law of heat conduction,


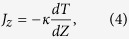


where *κ* is the thermal conductivity, 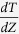 is the temperature gradient along z-direction and *J*_*z*_ is the heat current^22^.

## Results and Discussion

### Structural Characterization

#### Scanning Electron Microscopy

**Figure 2:**
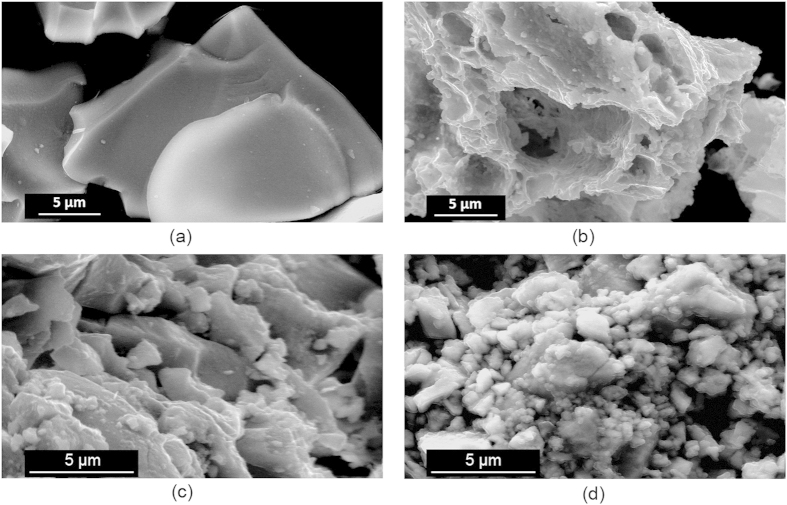
SEM images of: (**a**) 4YSZ particle; (**b**) DYZ particle; cross-sectional SEM images of pellets of: (**c**) 4YSZ; (**d**) DYZ.

The surface morphology of as-synthesized powders and pellets was characterized via SEM (Fig. 2), in order to study the influence of the microstructure on the thermodynamic properties. 4YSZ is composed of polyhedral particles with sharp edges, whereas DYZ particles exhibit more inter-particle pores. This could be due to the pyrolysis of the gel which consists of metallorganics and nitrate salts. The metallorganics tend to volatilize and combust, while nitrate salts appear to melt, forming hollow particles during combustion^28^. The formation of inter-particle pores could result in the exfoliation of small particles^29^. Moreover, the substitution concentration of ZrO_2_ influences the particle sizes: higher rare earth content tends to form smaller particles due to the fact that the rare earth atoms can increase particle brittleness^30^. The cross-sectional SEM images of the morphology of the pellets [Fig. 2(c,d)] show that DYZ exhibits relatively smaller particles as compared to those of 4YSZ.

#### X-ray Powder Diffraction

**Figure 3:**
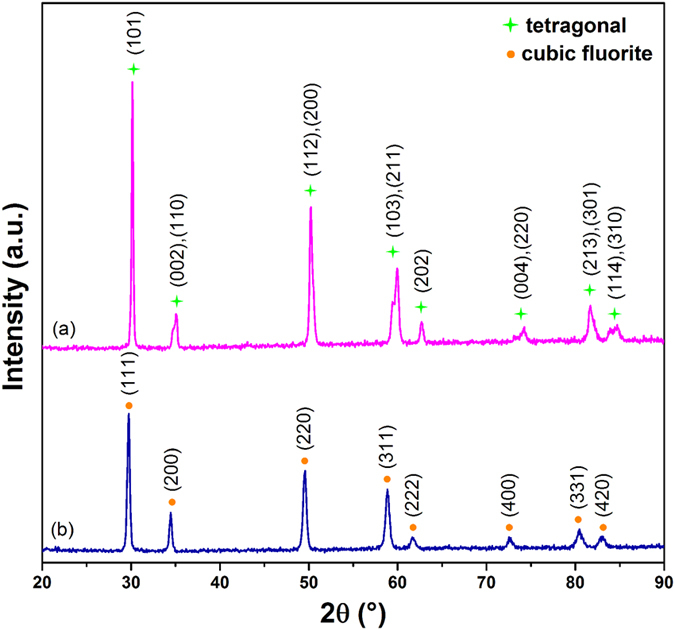
XRD patterns of samples sintered at 1200 °C for 2 hours for: (**a**) 4YSZ; (**b**) DYZ.

The phase composition was measured by XRD, as presented in Fig. 3, showing that 4YSZ consists of a tetragonal phase and DYZ consists of a cubic fluorite structure. This is confirmed from Raman analysis because both the tetragonal and cubic phases display relatively similar diffraction peaks in XRD patterns but have different vibration spectra.

The crystal size of the as-sintered powders was calculated based on line broadening using the Scherrer equation,


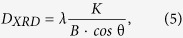


where *D* is the dimension of crystalline, *λ* is the wavelength (Cu-Kα), θ is the diffraction angle, *K* is the constant (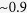) and *B* is the full-width at half maximum (FWHM)^31^. The FWHM can be determined based on,


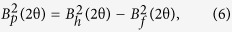


where *B*_*p*_(2θ) is the true half maximum width, *B*_*h*_(2θ) is the measured half maximum width of the sample and *B*_*f*_(2θ) the standard half maximum width^32^. The 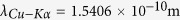, and the standard material is LaB_6_ 300. With the increase in the concentration of Dy_2_O_3_ and Y_2_O_3_, the average crystallite size decreases (Table 2). The presence of Dy^3+^ and Y^3+^ limits the grain growth of the powders.

#### Raman Analysis

**Figure 4:**
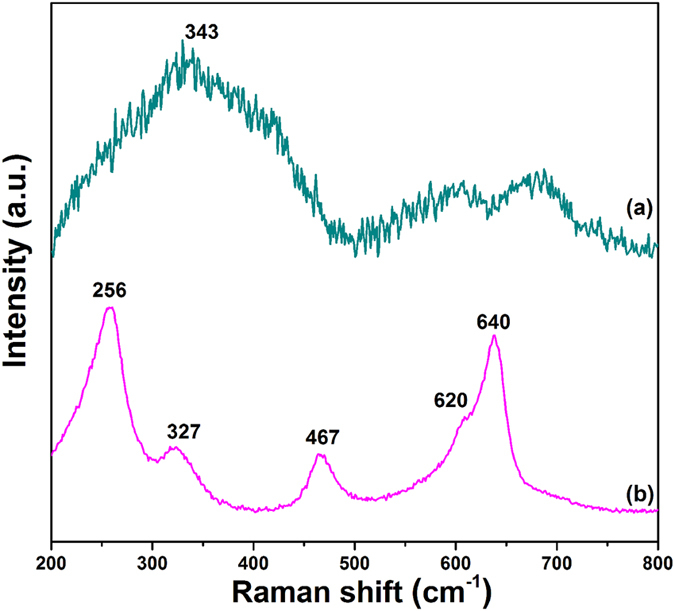
Raman spectra of 1200 °C sintered samples of: (**a**) DYZ (2-hour calcination); (**b**) 4YSZ (8-hour calcination).

The molecular symmetry was studied by Raman spectroscopy in order to determine the crystal structure due to the similar XRD peaks between the tetragonal structure and cubic structure, as presented in Fig. 4. In the Raman spectrum of 4YSZ, there are five active modes between 200 cm^−1^ and 800 cm^−1^. 256 cm^−1^ is the E_g_ stretching mode; 327 cm^−1^ indicates Zr-O B_1g_ bending mode; 467 cm^−1^ is Zr-O stretching E_g_ mode; 620 cm^−1^ is symmetric O-Zr-O A_1g_ stretching mode; and 640 cm^−1^ is asymmetric O-Zr-O E_g_ stretching mode^33^. The DYZ sample exhibits a broad-band Raman peak at 343 cm^−1^ which corresponds to the T_2g_ active mode, implying a defect fluorite structure^34^.

### Thermochemical Compatibility

**Figure 5:**
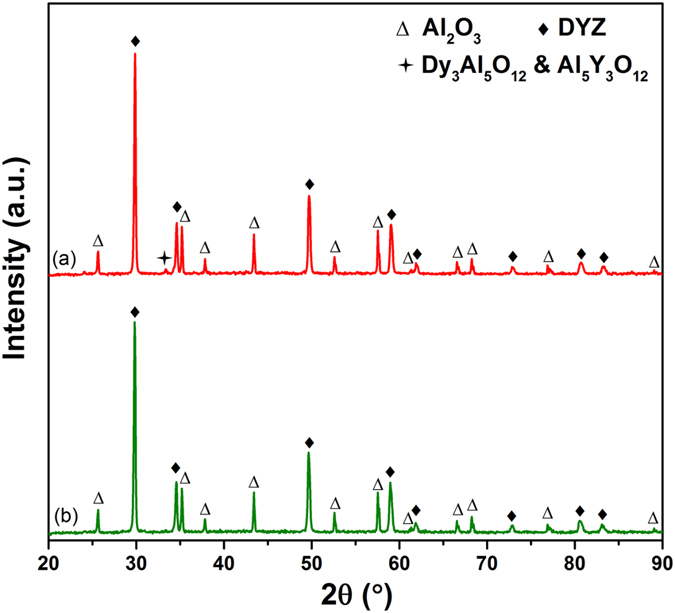
XRD patterns of DYZ-Al_2_O_3_ powder mixtures: (**a**) calcined at 1350 °C for 6 hours; (**b**) calcined at 1300 °C for 6 hours.

The thermochemical compatibility between Al_2_O_3_ and DYZ was studied via a solid-state reaction route in order to assess the high-temperature stability of DYZ to be used as a TBC material. The XRD patterns of 1300–1350 °C sintered mixed powders are shown in Fig. 5, implying that DYZ has thermochemical compatibility with Al_2_O_3_ up to 1300 °C. New diffraction peaks of a minor amount of Al_5_Y_3_O_12_ and Dy_3_Al_5_O_12_ are observed in the XRD pattern of 1350 °C sintered powder mixtures, suggesting that a solid-state reaction occurred at 1350 °C.

**Figure 6:**
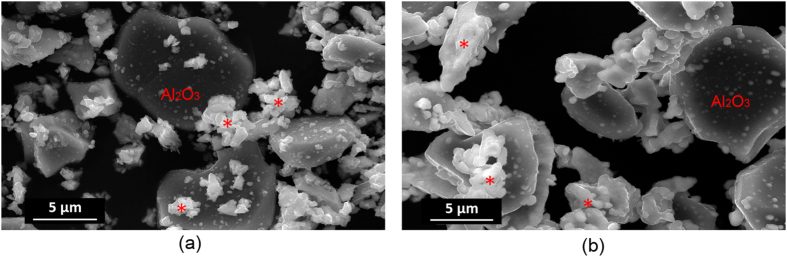
SEM images of powder mixtures: (**a**) ball milled DYZ and Al_2_O_3_ mixture; (**b**) DYZ and Al_2_O_3_ mixture sintered at 1350 °C for 6 hours (*represents DYZ particles).

The morphology of DYZ and Al_2_O_3_ powder mixtures before and after calcination was investigated using SEM, as depicted in Fig. 6. The DYZ and Al_2_O_3_ powder mixtures exhibit two morphologies, including Al_2_O_3_ with laminar structure and DYZ with polyhedral morphology. After calcination at 1350 °C, smaller DYZ particles are deposited on the surface of Al_2_O_3_, probably due to the reaction.

The reaction between Al_2_O_3_ and DYZ can be a result of the higher driving forces of the inter-diffusion of Dy^3+^ cations into Al_2_O_3_ crystals when the concentration of Dy^3+^ cations and Y^3+^ cations was 25 mol %, respectively. This process can be considered as a solid-state reaction which depends on the diffusion of Al^3+^, Dy^3+^ and Y^3+^, controlled by a parabolic rate law,


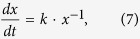


where *x* is the amount of reaction, *t* is time and *k* is the rate constant^35^. When the concentration of Dy^3+^ is high or the temperature of the reaction is increased, the reaction is more likely to occur.

**Figure 7:**
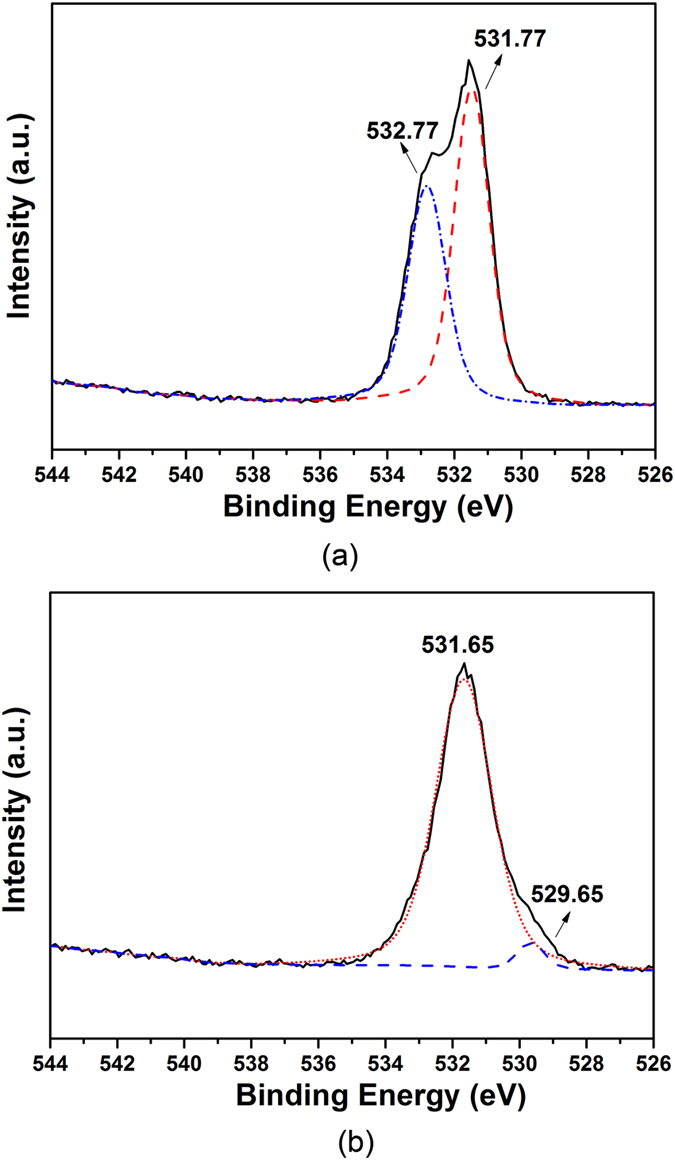
XPS spectra of O1s peaks of Al_2_O_3_-DYZ powder mixtures with 6-hour calcination at: (**a**) 1300 °C; (**b**) 1350 °C.

XPS measurements were conducted to investigate the local chemical environment and the binding energy of oxygen in Al_2_O_3_-DYZ powder mixtures after the calcination at 1300 °C and 1350 °C (Fig. 7). There are two O1s peaks at 532.77 eV and 531.77 eV after calcination at 1300 °C. The double peaks in Fig. 7(a) could represent the O1s peak in DYZ and the O1s peak in Al_2_O_3_ due to the fact that the local chemical environment of the oxygen in DYZ and Al_2_O_3_ is different^36^. However, after the calcination at 1350 °C, the double peaks become one broad peak at 531.65 eV, which could be explained by the improved similarity of the local chemical environment in the 1350 °C sintered Al_2_O_3_-DYZ powder mixture. A low-intensity O1s peak at 529.65 eV appears, probably resulting from the carbon-oxygen compound that is absorbed on the sample surface^37^. Moreover, this low-intensity O1s peak could also be due to the formation of ZrO_2_ between Al_2_O_3_ and DYZ, which has a relatively lower binding energy for O1s at 529.9 eV^36^. This agrees well with the XRD results, which show that after sintering at 1350 °C the solid-state reaction between Al_2_O_3_ and DYZ occurs and new phases form when ZrO_2_ is an additional product. The amount of new phases is low and the reaction occurs primarily at the interfaces between Al_2_O_3_ and DYZ, hence resulting in the change of the binding energy of the O1s peak.

### Thermal Conductivity

The thermal conductivity of the pellets was measured using the TCi thermal conductivity analyser at room temperature. The porosity of the sintered pellets was determined using the density equation,


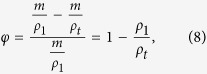


where *φ* is the porosity, *m* is the mass of the sample, *ρ*_1_ is the measured density and *ρ*_*t*_ is the theoretical density. The bulk thermal conductivity is modified considering the presence of pores within the sintered bulk pellets. These pores can scatter phonons and reduce the mean free path of these phonons, so to determine the intrinsic thermal conductivity, fully dense thermal conductivity needs to be calculated based on equation (1). The specific heat can be derived from,


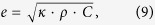


where *e* is the effusivity, *κ* is the thermal conductivity, *ρ* is the density and *C* is the specific heat capacity^38^. The bulk modulus is determined by,


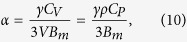


where *C*_*V*_ is the molar isochoric heat capacity, *V* is the molar volume, *C*_*P*_ is the isobaric heat capacity, *ρ* is the density, *B*_*m*_ is the bulk modulus, *α* is the coefficient of thermal expansion and *γ* is the Grüneisen constant^39^. The coefficient of thermal expansion at 900 °C is used for the calculation of the bulk modulus from equation (10). The Young’s modulus is calculated via,


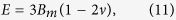


where *E* is the Young’s modulus, *ν* is the Poisson’s ratio and *B*_*m*_ is the bulk modulus^40^. The minimum thermal conductivity can be calculated by,


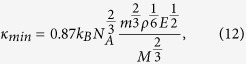


where *E* is the Young’s modulus, *M* is the molecular weight, *m* is the number of atoms per molecular formula, *k*_*B*_ is Boltzmann’s constant and *N*_*A*_ is Avogadro’s number^41^.

Table 3 depicts the corrected thermal conductivity of DYZ and 4YSZ bulk samples and the minimum thermal conductivity via Clarke’s model^5^. In comparison with the measured values of 4YSZ using the same instrument, the thermal conductivity of DYZ is lower. The thermal conductivity of DYZ is lower than that of Dy_2_Zr_2_O_7_ which is reported by Qu *et al.*^14^. Clarke’s model is based on the Debye equation and the minimum phonon mean free path is assumed to be the cube root of the volume of a molecule^5^. The mean phonon velocity is approximated to be the mean acoustic velocity, which ignores the optical phonon modes, resulting in an underestimation of the thermal conductivity.

Higher doping content could result in enhanced mass differences and ionic radii misfit and more oxygen vacancies within the lattice; therefore the phonon scattering is stronger. The addition of Dy^3+^ and Y^3+^ into the ZrO_2_ lattice creates oxygen vacancies to maintain the charge balance. Based on the defect chemistry and Kroger-Vink notation, the formation of Dy^3+^substitution defect can be expressed as,


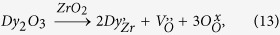


where 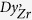 indicates Dy^3+^ cation taking the Zr^4+^ cation site with a single negative charge, 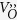 is an oxygen vacancy with double positive charges, and 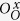 represents O^2−^ anion on the oxygen site with neutral charge^42^.

In ionic oxide ceramics, two types of phonon behaviour influence the thermal conductivity, namely phonon-phonon scattering and phonon-defect scattering. In the phonon-defect scattering process, the scattering coefficient (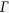) for 4YSZ and DYZ can be determined using equation (11)–(13),


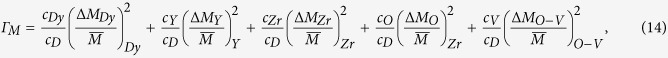


where *Γ*_*M*_ is the defect scattering coefficient due to mass misfit, 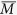 is the average atomic weight in a unit cell, *C*_*Y*_, *C*_*Dy*_, *C*_*Zr*_, *C*_*O*_, *C*_*V*_, and *C*_*D*_ are the content of Y^3+^, Dy^3+^, Zr^4+^, O^2−^, oxygen vacancy, and overall defects, Δ*M*_*Dy*_, Δ*M*_*Y*_ and Δ*M*_*Zr*_ are the atomic mass differences between the average atomic weight of the cation site (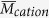) and Dy/Y/Zr, and Δ*M*_*O*_ and Δ*M*_*O-V*_ are the atomic mass differences between the anion site and O^2−^/oxygen vacancy. Specifically, 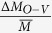 is equal to 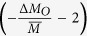 due to the fact that the number of broken bonds of an oxygen vacancy is twice as high as that per atom^43^.


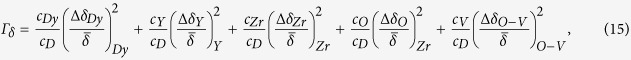


where *Γ*_*δ*_ is the defect scattering coefficient due to radius misfit, 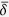 is the average radius in a unit cell, 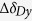, 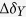, and 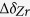 are the radius differences between the average radius of the cation site (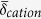) and Dy/Y/Zr, respectively, and 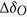 and 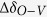 are the radius differences between anion site and O^2−^/oxygen vacancy. The effective radius of an oxygen vacancy is assumed to be zero^44^.


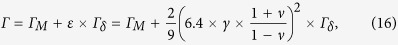


where *Γ* is the total defect scattering coefficient, *ε* is the strain field factor, *γ* is the Grüneisen parameter and *ν* is Poisson’s ratio^45^. The Grüneisen parameter *γ* for 4YSZ is 1.40^46^ and the Poisson’s ratio for 4YSZ is 0.20^47^. The Grüneisen parameter *γ* for DYZ is assumed to be 1.54^45^, estimated according to La_2_Zr_2_O_7_, and the Poisson’s ratio is 0.294^34^, estimated based on Dy_2_Zr_2_O_7_.

Table 4 shows the scattering coefficient for DYZ and 4YSZ, suggesting that the scattering coefficient of 4YSZ is lower than that of DYZ. The atomic weight of Dy (162.5) is much heavier than that of Y (88.906), resulting in the higher scattering of phonon in DYZ as compared with that in 4YSZ due to the fact that the mean free path of the phonon is inversely proportional to 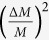
48.

### Predicted Thermodynamic Properties

**Figure 8:**
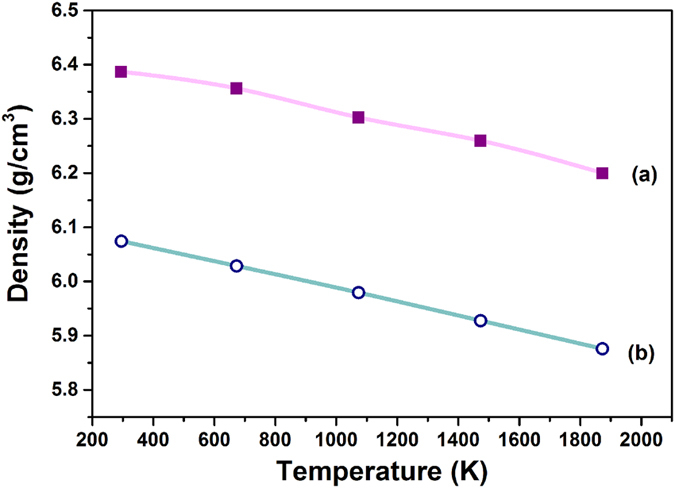
Predicted density of: (**a**) DYZ; (**b**) 4YSZ.

The thermal conductivity of DYZ and 4YSZ has been calculated via MD simulation in order to investigate the intrinsic property of the perfect lattice from room temperature to high temperatures. The reduction of thermal conductivity can be achieved by creating a novel composition such as a solid solution because dopants and the corresponding oxygen vacancies can reduce the intrinsic thermal conductivity via scattering lattice waves^49^. At 295 K, the density of 4YSZ calculated from the equilibrated crystal structure is 6.0742 g/cm^3^, which agrees well with the published data of 6.0–6.1 g/cm^3^ 50,51, and the calculated density of DYZ (6.3868 g/cm^3^) is in strong agreement with the experimental data (6.4093 g/cm^3^). Hence, the potential parameters are sufficient to generate correct lattice structure of (Dy, Y)-ZrO_2_ in the simulation. The density of DYZ and 4YSZ from 295 K to 1874 K is presented in Fig. 8, which shows that DYZ has a relatively higher density as compared with 4YSZ. The decrease in density with the increase in temperature is due to the thermal expansion of the lattice.

The calculated thermal conductivity of DYZ and 4YSZ exhibits a decreasing trend with the increase in temperature from 295 K to 1873 K, as depicted in Table 5. This can be explained by the Umklapp phonon-phonon scattering process which dominates the heat conduction of ionic crystals at high temperature^45^. Between 295 K and 1873 K, the thermal conductivity of 4YSZ decreases by 34% which is higher than that of DYZ (11%). The influence of the Umklapp scattering process against temperature on the decrease of the thermal conductivity of 4YSZ is higher, as compared with that of DYZ. Moreover, the calculated thermal conductivity of DYZ is lower than that of 4YSZ due to the enhanced phonon-defect scattering process. The incorporation of every two Dy^3+^ cations into the ZrO_2_ lattice creates one oxygen vacancy in order to maintain the charge neutrality. This oxygen vacancy, the mass difference, and the ionic radii difference between Dy^3+^ and Zr^4+^, Y^3+^ and Zr^4+^ decrease the phonon mean free path and thermal conductivity^52^.

The thermal conductivity of 4YSZ predicted from the simulation is higher than that measured in the experiment (2.3–3.0 W/m·K)^1^,^11^,^53^,^54^ due to the fact that in the experiment there are defects and boundaries which can reduce the thermal conductivity. These defects and boundaries include grain boundaries, interfaces between dissimilar phases, defect clusters, micro-cracks and nano-pores^41^,^55^,^56^. The model is based on a single crystal without the influence of such defects, partly resulting in higher thermal conductivity. Moreover, as in the model reported by Schelling and Phillpot, at low temperature the relatively high thermal conductivity can be partly due to the high speed of sound in the model and the high-frequency vibrational modes which are different from the experimental work and lead to an increase of thermal conductivity^21^. This is because MD simulation is based on the assumption of Newton’s equation of motion, ignoring the detail of the electronic structure of particles^17^.

In addition, finite-size effects could result in the discrepancy of the thermal conductivity of DYZ and 4YSZ between the experiment and MD simulation. The thermal conductivity could be influenced by the length of the simulation cell L_Z_, and 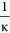 is proportional to 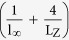 where 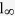 is the phonon mean free path of an infinite system^22^. DYZ has a lower thermal conductivity, indicating that the phonon mean free path of DYZ is smaller than that of 4YSZ, especially at a lower temperature. However, the length of the simulation cell is identical for DYZ and 4YSZ, thus resulting in a larger deviation of the calculated thermal conductivity for 4YSZ than for DYZ. Moreover, it is reported by Clarke that phonons reach a minimum mean free path at high temperatures, and this is comparable with two neighbouring atoms in a lattice^5^. Thus, the thermal conductivity of DYZ and 4YSZ is more similar with each other at high temperatures than at room temperature.

## Conclusions

In summary, fluorite DYZ powders and tetragonal 4YSZ powders have been synthesized via a sol-gel route. The particles exhibit polyhedral morphology with sharp edges and pores. The thermal conductivity of DYZ is lower than that of 4YSZ and the phonon-defect scattering of DYZ is stronger than that of 4YSZ. Theoretical thermal conductivity of DYZ is lower than that of 4YSZ, and the density of DYZ is higher than that of 4YSZ from 295 K to 1873 K. The influence of doping Dy^3+^ and Y^3+^ cations on the calculated thermal conductivity of ZrO_2_ via MD simulation is in good agreement with the experimental measurement. At 1300 °C, there is no reaction between DYZ and Al_2_O_3_, whereas a minor amount of Al_5_Y_3_O_12_ and Dy_3_Al_5_O_12_ formed due to calcination at 1350 °C. It is discovered that DYZ exhibits lower thermal conductivity and has the potential to be used up to 1300 °C as a new ceramic TBC material.

## Additional Information

**How to cite this article**: Qu, L. *et al.* An Atomistic-Scale Study for Thermal Conductivity and Thermochemical Compatibility in (DyY)Zr_2_O_7_ Combining an Experimental Approach with Theoretical Calculation. *Sci. Rep.* **6**, 21232; doi: 10.1038/srep21232 (2016).

## Figures and Tables

**Table 1:** Interatomic potential used in the simulation.

Species	*A* (eV)	*ρ* (Å)	*C* (eV·Å^6^)	Ref
Y^3+^-O^2−^	1366.35	0.348	19.6	22
Zr^4+^-O^2−^	1502.11	0.345	5.1	22
O^2−^-O^2−^	9547.96	0.224	32	22
Dy^3+^-O^2−^	1807.84	0.3393	18.77	57

**Table 2:** Average crystallite size in 4YSZ (101) plane and DYZ (111) plane after calcination at 950 °C for 3 hours.

	4YSZ	DYZ
Average crystal size (nm)	22.51 ± 2.56	10.62 ± 0.78

**Table 3:** Thermal conductivity of dense pelletized powders.

	4YSZ	DYZ
Effusivity (W· 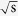 /m^2^·K)	1410.70 ± 66.87	1047.01 ± 44.37
Specific heat (J/kg/K)	329.38 ± 7.82	422.34 ± 42.53
Thermal conductivity (W/m·K)	2.3517 ± 0.2610	1.6727 ± 0.2861
Coefficient of thermal expansion ( 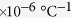 )	12.71	13.01
Young’s modulus (GPa)	131	132
Density (g/cm^3^)	6.0339	6.4093
*κ*_*min*_ (W/m·K)	1.1075	0.9933

**Table 4:** The scattering coefficient of 4YSZ and DYZ.

	Γ_*M*_	Γ_*δ*_	*ε*	Γ
4YSZ	0.073	0.018	40	0.78
DYZ	0.58	0.11	60	7.22

**Table 5:** Predicted thermal conductivity via non-equilibrium MD simulation.

	4YSZ	DYZ
295 K	6.1408 ± 0.3634	2.4295 ± 0.1290
673 K	5.1373 ± 0.4962	2.3161 ± 0.1124
1073 K	4.8565 ± 0.6986	2.2172 ± 0.3472
1473 K	4.4404 ± 0.4010	2.0492 ± 0.3880
1873 K	4.0411 ± 0.2539	2.0531 ± 0.5538
